# NOP14 suppresses breast cancer progression by inhibiting NRIP1/Wnt/β-catenin pathway

**DOI:** 10.18632/oncotarget.4573

**Published:** 2015-07-13

**Authors:** Jin-Ju Lei, Rou-Jun Peng, Bo-Hua Kuang, Zhong-Yu Yuan, Tao Qin, Wen-Sheng Liu, Yun-Miao Guo, Hui-Qiong Han, Yi-Fan Lian, Cheng-Cheng Deng, Hao-Jiong Zhang, Li-Zhen Chen, Qi-Sheng Feng, Miao Xu, Lin Feng, Jin-Xin Bei, Yi-Xin Zeng

**Affiliations:** ^1^ Department of Experimental Research, Sun Yat-sen University Cancer Center, State Key Laboratory Oncology in South China, Collaborative Innovation Center of Cancer Medicine, Guangzhou, China; ^2^ Department of Medical Oncology, Sun Yat-sen University Cancer Center, State Key Laboratory Oncology in South China, Collaborative Innovation Center of Cancer Medicine, Guangzhou, China; ^3^ Peking Union Medical College, Beijing, China

**Keywords:** NOP14, breast cancer, NRIP1, Wnt/β-catenin pathway

## Abstract

NOP14, which is functionally conserved among eukaryotes, has been implicated in cancer development. Here, we show that NOP14 is poorly expressed in breast cancer cells and invasive breast cancer tissues. *In vivo* and *in vitro* studies indicated that NOP14 suppressed the tumorigenesis and metastasis of breast cancer cells. Further investigations revealed that NOP14 enhanced ERα expression and inhibited the Wnt/β-catenin pathway by up-regulating NRIP1 expression. Survival analysis indicated that low NOP14 expression was significantly associated with poor overall survival (*P* = 0.0006) and disease-free survival (*P* = 0.0007), suggesting that NOP14 is a potential prognostic factor in breast cancer. Taken together, our findings reveal that NOP14 may suppress breast cancer progression and provide new insights into the development of targeted therapeutic agents for breast cancer.

## INTRODUCTION

Breast cancer is the most common cancer that occurs in women worldwide and is the second cause of cancer-related mortality in women, accounting for 15% of all cancer deaths [1, 2]. Metastasis is known to be the leading factor for the resultant mortality and poor prognosis for breast cancer patients. Therefore, it is essential to reveal the mechanisms involved in breast cancer progression, so as to identify useful prognosis biomarkers and new therapeutic targets.

*NOP14*, which function is well conserved among eukaryotes, is a stress-responsive gene that is required for 18S rRNA maturation and 40S ribosome production [3]. Results of a conventional UV cross linking assay carried out in HeLa cells suggested that NOP14 is likely an RNA binding protein that determines RNA fate from synthesis to decay [4]. Recent studies have suggested that NOP14 may be involved in cancer development. In prostate cancer cells, NOP14 is a target gene of the polycomb repressive complex that has been shown to play a critical role in neoplastic progression [5, 6]. Moreover, NOP14 is one of the proteins that interact with PAXIP1, which contains tandem breast cancer carboxy-terminal domains and regulates multiple aspects of the cellular response to DNA damage, such as cell survival and differentiation [7–11]. More recently, a substantial number of somatic NOP14 mutations have been identified in the liver metastases derived from pancreatic ductal adenocarcinoma [12]. Subsequent functional study revealed that NOP14 overexpression was able to promote pancreatic cancer cell proliferation and metastasis both *in vitro* and *in vivo* [13].

Based on these studies, we attempted to investigate the role of NOP14 in breast cancer development. The expression patterns of NOP14 in human breast cancer cell lines and tissues were examined, and the functional role of NOP14 in breast tumorigenesis were characterized both *in vitro* and *in vivo*; moreover, the underlying mechanism by which NOP14 suppresses the development of breast cancer was investigated. Finally, the NOP14 expression with respect to prognosis of breast cancer patients was also evaluated.

## RESULTS

### NOP14 expression patterns in breast cell lines and tissues

**Figure 1:**
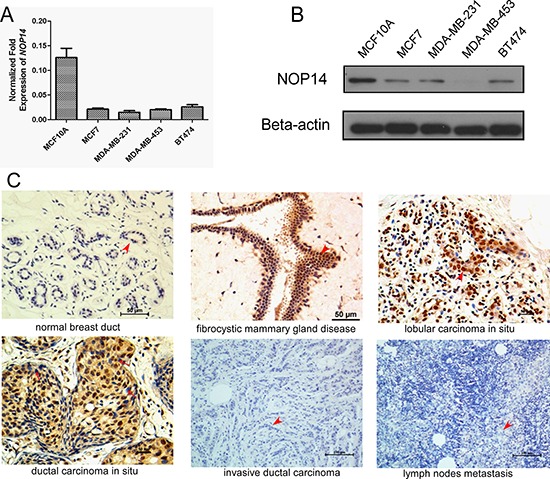
NOP14 expression in different breast cell lines and tissues **A.** Relative mRNA expression of NOP14 in the immortalized fibrocystic breast cell lines (MCF10A) and four breast cancer cell lines (MCF-7, MDA-MB-231, MDA-MB-453 and BT474). **B.** Western blot of NOP14 expression in the above cell lines; and β-actin was the loading control. **C.** Representative images for IHC staining of NOP14 expression in different breast tissues.

NOP14 expression in breast cell lines was determined at the transcription level by quantitative real-time polymerase chain reaction (qPCR) and at the protein level by western blot (WB) analysis. High levels of NOP14 mRNA and protein were observed in the fibrocystic breast cell line MCF10A; whereas the levels of NOP14 mRNA and protein were low in the four breast cancer cell lines including MCF7, MDA-MB-231, MDA-MB-453 and BT474 (Figure 1A and 1B).

We further analyzed NOP14 expression levels in human mammary biopsies by immunohistochemical (IHC) staining. Compared with the normal mammary tissue, strong NOP14 expression was detected in the fibrocystic tissue with atypical ductal hyperplasia (ADH), as well as in lobular and ductal carcinomas *in situ* (Figure 1C). Strikingly, NOP14 expression could barely be detected in 254 invasive breast cancer tissues and 63 paired metastatic lymph nodes (Figure 1C, Figure S1 and Table S1). These results indicated that NOP14 levels correlated reversely with the malignancy of human breast cancer, which was high in ADH and primary cancer but low in the advanced breast cancer tissues.

### NOP14 overexpression suppresses tumorigenesis and metastasis

**Figure 2:**
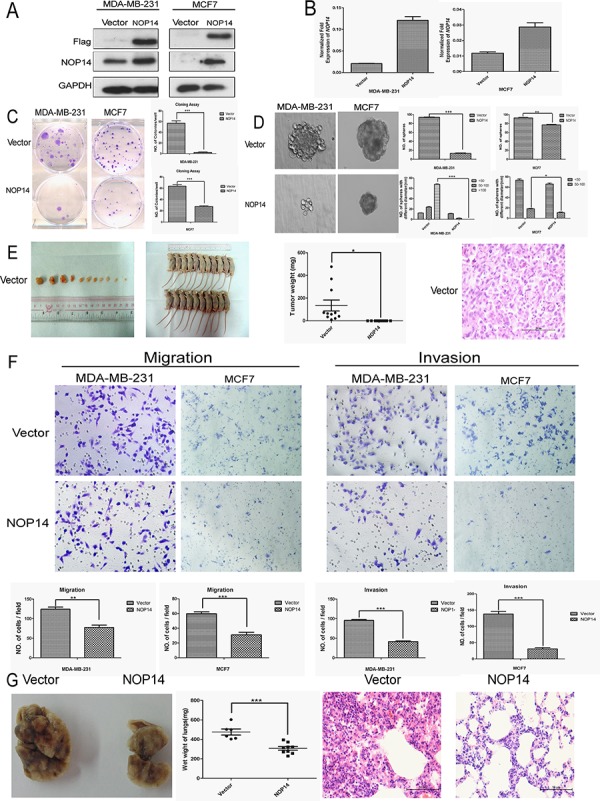
NOP14 overexpression inhibits tumorigenesis and metastasis of breast cancer cells **A.** Western blot analysis showing the exogenous expression of recombinant Flag-NOP14 protein, using primary antibodies against Flag and NOP14, respectively; and GAPDH expression was a loading control. **B.** qPCR showing mRNA transcription in stable cell lines with or without NOP14 constructs. **C.** Representative images of reduced foci formation in plate cultures. Quantitative analysis of foci numbers are shown in the right panel. Values reflect the mean ± 3SD of at least three independent experiments. **D.** Representative images of the decreased sphere forming ability of NOP14-expressing cells. The results are summarized as the mean ± 3SD of three independent experiments in the right panel. **E.** Tumor formation in nude mice demonstrates decreased *in vivo* tumorigenicity in NOP14-expressing cells (no tumor formed in the NOP14 overexpression group) compared with empty vector-transfected cells. **F.** Transwell assays and Matrigel invasion assays with representative image as upper panel and statistical results as the mean ± 3SD of three independent experiments in the lower panel. **G.** *In vivo* metastasis results with cell lines with empty vector or NOP14 construct. Panel for the left to the right are representative images of lung metastasis, wet weight and HE staining, respectively. (*P < 0.05, **P < 0.01, ***P < 0.001, independent Student's *t* test).

To identify the role of NOP14 in breast cancer development, exogenous human NOP14 gene was transfected into MDA-MB-231 and MCF-7 breast cancer cells (Figure 2A and 2B). Transfection of NOP14 cDNA significantly reduced the colony (Figure 2C) and sphere formation of breast cancer cells compared with those transfected with control vector (Figure 2D). However, no significant effects of NOP14 overexpression on cell proliferation were observed in the two cell lines according to the Cell Counting Kit-8 (CCK-8) assay results (Figure S2A and S2B). As CD44^+^/CD24^−/low^ is the classic stem cell marker in breast cancer [14, 15], and approximately 99% of MDA-MB-231 cells are CD44^+^ [16], we focused on the level of CD24 in MDA-MB-231 cells with or without NOP14 overexpression. Indeed, higher CD24 expression, which represents lower stem cell content, was observed in NOP14-overexpressing cells than that in the control cells (Figure S3A and S3B). Moreover, all the nude mice developed tumor after subcutaneous inoculation with MDA-MB-231 cells carrying the empty vector (*n* = 11), while no tumors formation were observed in mice inoculated with MDA-MB-231 cells overexpressing NOP14 (*n* = 10) (Figure 2E), suggesting that up-regulation of NOP14 inhibits tumorigenesis in xenograft mouse models of breast cancer.

Transwell assays of MDA-MB-231 and MCF-7 cells showed that NOP14 overexpression resulted in much fewer migrated cells compared with the control groups (Figure 2F). Similarly, invasion assays with Matrigel also indicated that NOP14 overexpression inhibited cell invasion in both MDA-MB-231 and MCF-7 cells (Figure 2F). Next we determined the role of NOP14 in breast cancer metastasis *in vivo* by intravenous injection of MDA-MB-231 cells via the tail veins. Compared to the control mice injected with MDA-MB-231 cells expressing empty vector, the mice injected with NOP14-overexpressing MDA-MB-231 cells had significantly decreased wet weight of the lungs and less metastasis lesions, as reflected by the invasive areas in the HE staining assay (Figure 2G).

### NOP14 silencing promotes tumorigenesis and metastasis

**Figure 3:**
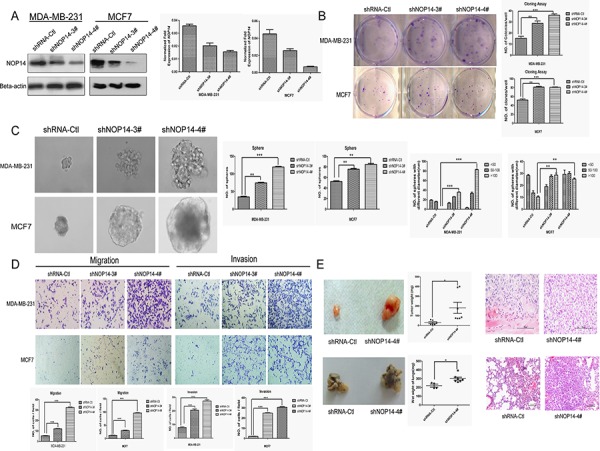
NOP14 silencing increases tumorigenicity and metastasis **A.** Two shRNAs (shNOP14–3# and shNOP14–4#) directed against NOP14 effectively decreased NOP14 expression as detected by qPCR and western blot. Scrambled shRNA Control (shRNA-Ctl) and β-actin were used as controls. **B.** Representative images of increased foci formation in plate cultures. Quantitative analysis of foci numbers are shown in the right panel. Values are reflected as the mean ± 3SD of at least three independent experiments. **C.** Representative images of the increased sphere-forming ability of NOP14-silenced cells. The results are summarized as the mean ± 3SD of three independent experiments in the right panel. **D.** Transwell migration and invasion assays for which the results are summarized as the mean ± 3SD of three independent experiments as shown in the lower panel. **E.** Representative images of increased tumor formation in nude mice (upper), lung metastasis (lower), wet weight and HE staining shown in the right panel. (*P < 0.05, **P < 0.01, ***P < 0.001, independent Student's *t* test).

By using targeted short hairpin RNAs (shRNAs), we down-regulated NOP14 levels in MDA-MB-231 and MCF-7 cell lines and the knockdown efficiencies were demonstrated by Western blot and qPCR assays (Figure 3A). Colony and sphere formation assays indicated that knockdown of NOP14 enhanced colony formation (Figure 3B) and sphere formation (Figure 3C) in both MDA-MB-231 and MCF-7 cells; however, no significant differences in the growth rates were observed between the control and NOP14 knockdown cells (Figure S2C and D). Besides, lower CD24 expression was detected in the NOP14-silenced cells than that in the control cells (Figure S3C and S3D). Xenograft model showed that knockdown of NOP14 by shRNA #4 (7 of 7 mice) resulted in larger and heavier tumor formation than that of the mock transfected (6 of 7 mice) (Figure 3E).

Transwell assays showed that the MDA-MB-231 cells stably expressing NOP14 shRNA had substantially higher migratory abilities compared with the control cells. Moreover, Matrigel invasion assays revealed that inhibition of NOP14 expression promoted the invasion abilities of MDA-MB-231 and MCF-7 cells (Figure 3D). Moreover, in contrast to the control mice that were injected with MDA-MB-231 cells carrying non-targeting shRNA, mice intravenously injected with MDA-MB-231 cells stably expressing NOP14 shRNA displayed much heavier weight of wet lung, as well as more aggressive metastasis as reflected by the larger invasive area in the HE staining assay (Figure 3E).

### NOP14 inhibits migration and invasion *via* NRIP1

**Figure 4:**
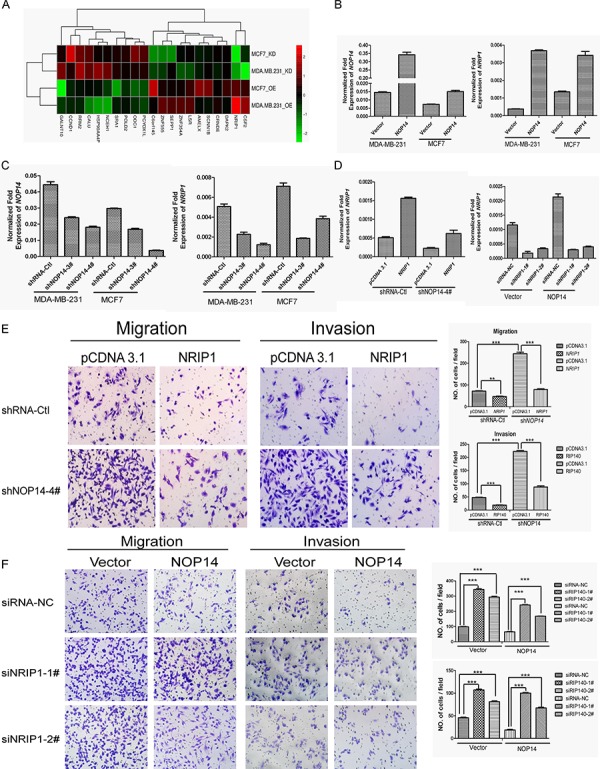
NOP14 inhibits migration and invasion via NRIP1 **A.** Gene expression array results for NOP14-overexpressed and NOP14-knockdown cells compared with corresponding control cells. OE, overexpression group; KD, knockdown group. **B, C.** qPCR of *NOP14* and *NRIP1* in NOP14 stable and control cell lines. **D.** qPCR of *NRIP1* in transiently transfected cells. **E, F.** Transwell assays. The results are summarized as the mean ± 3SD of three independent experiments as shown in the right panel (***P < 0.001, independent Student's *t* test).

To uncover the mechanisms underlying the inhibition of NOP14 on breast cancer progression, genome-wide gene expression assays were conducted in two pairs of the NOP14 overexpression and the control cell lines, and two pairs of NOP14 knockdown and the control cell lines (Figure 4A and Figure S4A). By comparing the relative expression ratios of 28, 869 genes, we discovered that *NRIP1* was consistently upregulated in NOP14-overexpressing cells and down-regulated in NOP14-knockdown cells (Table S2), which was further validated by qPCR assays (Figure 4B, 4C and Figure S4B).

To further characterize the relationship between NRIP1 and NOP14, NRIP1 was transiently transfected into NOP14 knockdown cells (MDA-MB-231-shNOP14–4#) or control cells (MDA-MB-231-shRNA-Ctl) (Figure 4D). Transwell assays showed that introducing of NRIP1 to NOP14-depleted cells significantly suppressed the high migration and invasion capabilities caused by NOP14 knockdown (Figure 4E). Likewise, introducing NRIP1 to control cells also exhibited an inhibitory effect on migration and invasion. By contrast, transwell assays indicated that depletion of NRIP1 by siRNAs in cells with or without NOP14 overexpression promoted the migration and invasion of breast cancer cells. Moreover, the inhibitory effects of NOP14 on the migration and invasion of MDA-MB-231 cells were reversed by NRIP1 depletion (Figure 4F). These results suggested that the inhibitory effects of NOP14 in breast cancer progression were NRIP1-dependent.

### NOP14 suppresses the Wnt/β-catenin pathway

**Figure 5:**
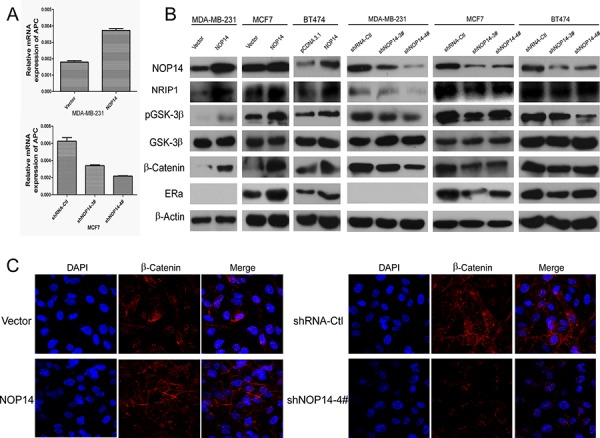
NOP14 inhibits the Wnt/β-catenin pathway **A.** qPCR of APC in NOP14-expressing and NOP14-silenced cells. **B.** Western blot analysis of NOP14-expressing and NOP14-silenced cells compared with corresponding control cells. **C.** IF of β-catenin in NOP14-expressing and NOP14-silenced cells compared with corresponding control cells. All the results were replicated at least three times.

Further attempt to evaluate the involvement of NOP14 in the Wnt/β-catenin signaling pathway was inspired by a recent finding that NRIP1 inhibits Wnt pathway *via* up-regulating APC [17]. qPCR assays showed that APC mRNA expression was increased by overexpressing NOP14, while APC mRNA was decreased by depletion of NOP14 (Figure 5A), suggesting a positive correlation between APC and NOP14 expression levels. Moreover, Western blot analysis of the proteins participating in the Wnt/β-catenin pathway showed that the levels of NRIP1, β-catenin and GSK-3β phosphorylation were elevated by NOP14-overexpressing in three breast cancer cell lines (MDA-MB-231: ER-negative; MCF7 and BT474: ER-positive). Meanwhile, an increase of estrogen receptor α (ERα) was also observed in MCF-7 and BT474 cells with overexpressing NOP14 (Figure 5B). Consistently, depletion of NOP14 resulted in reduced expression of the aforementioned proteins (Figure 5B). In addition, immunofluorescence (IF) staining showed that although β-catenin distributed throughout the cells, it accumulated primarily on the membranes of MDA-MB-231 cells upon NOP14 overexpression. On the contrary, β-catenin localized primarily at the nuclei of cells carrying NOP14 shRNA (Figure 5B). These results indicated that NOP14 could assemble β-catenin on the membranes of breast cancer cells, and prevent its nucleus translocation and the following activation.

### NOP14 expression is a prognostic marker for breast cancer

**Figure 6:**
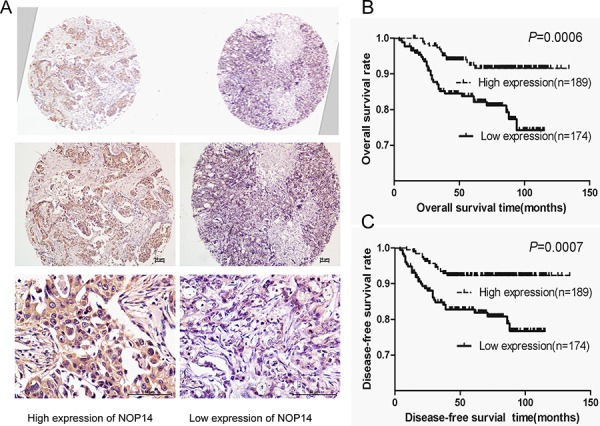
NOP14 expression in breast cancer TMA **A.** Representative IHC images of two adjacent tissues on a single slide under different magnifications. **B** and **C.** Kaplan-Meier analysis of NOP14 expression and overall survival rate (B) and disease-free survival rate (C) for breast cancer patients.

Breast cancer cases were classified as two groups by their NOP14 expression levels in their pathological sections (Figure 6A). Association analyses showed that the expression of both ER (*P* = 0.026) and progesterone receptor (PR; *P* = 0.01) positively correlated with NOP14 level in breast cancers (Table 1). Moreover, Kaplan-Meier analysis revealed that higher NOP14 expression was significantly associated with better overall survival (*P* = 0.0006, Figure 6B) and disease-free survival (*P* = 0.0007, Figure 6C), respectively. Furthermore, multivariate Cox regression analysis showed that NOP14 expression is an independent prognostic factor for overall survival rate (*P* = 0.001) and disease-free survival rate (*P* = 0.003) (Table 2).

**Table 1:** Clinical characteristics of breast cancer patients with TMA

Features	NOP14 low expression	NOP14 high expression	*P*-value
Age(years)			
≤40	35	42	0.7
>40	139	147	
Tumor grade			
1	30	33	0.971
2	96	102	
3	48	54	
Tumor size			
T1 (≤2 cm)	49	57	0.906
T2 (>2 cm, ≤5 cm)	112	119	
T3 (>5 cm)	13	13	
Lymph node status			
0	82	95	0.402
1–3	48	49	
4–9	23	31	
>9	21	14	
Stage			
I-II	126	135	0.907
III	48	54	
ER status			
Positive	63	91	**0.026**
Negative	111	98	
PR status			
Positive	57	87	**0.01**
Negative	117	102	
HER2 status			
Positive	50	58	0.731
Negative	124	131	
Ki67			
>14%	87	98	0.753
≤14%	87	91	
Menstrual status			
Premenopausal	72	68	0.332
Postmenopausal	102	121	

ER, estrogen receptor; PR, progesterone receptor;HER2, human epidermal growth factor receptor 2

**Table 2:** Univariate and multivariate analyses of prognostic factors

Variable	Univariate				Multivariate			
	OS		DFS		OS		DFS	
	OR (95% CI)	*P* value	OR (95% CI)	*P* value	OR (95% CI)	*P* value	OR (95% CI)	*P* value
NOP14 (High vs Low)	0.35 (0.19–0.66)	**0.001**	0.35 (0.19–0.66)	**0.001**	0.34 (0.18–0.66)	**0.001**	0.38 (0.20–0.73)	**0.003**
Age (>40 vs ≤40)	1.60 (0.85–3.00)	0.142	1.70 (0.91–3.18)	0.99	0.52 (0.23–1.17)	0.12	0.48 (0.21–1.09)	0.078
Tumor size								
T2 vs T1	2.55 (1.07–6.09)	**0.035**	2.54 (1.07–6.07)	**0.036**	1.23 (0.41–3.67)	0.460	1.23 (0.41–3.69)	0.713
T3 vs T2	5.15 (1.73–15.35)	**0.003**	5.09 (1.71–15.15)	**0.003**	2.30 (0.70–11.11)	0.14	2.56 (0.56–9.07)	0.252
Lymph nodes								
0–3 vs 0	1.86 (0.77–4.46)	0.167	1.87 (0.78–4.50)	0.167	1.80 (0.68–4.74)	0.23	1.74 (0.66–4.60)	0.25
4–9 vs 0	4.61 (2.02–10.53)	**2.823 E-4**	4.75 (2.08–10.84)	**2.147 E-4**	33.26 (4.48–247.06)	**0.0006**	29.53 (4.09–213.11)	**0.0007**
≥10 vs 0	7.32 (3.208–16.71)	**2.263 E-6**	7.46 (3.27–17.02)	**1.825 E-6**	65.17 (7.23–587.35)	**0.0001**	54.05 (6.21–470.14)	**0.0003**
TNM stage (III vsI-II)	3.27 (1.82–5.88)	**6.54 E-5**	3.32 (1.85–5.88)	**5.14 E-5**				
Tumor grade								
2 vs 1	3.11 (0.73–13.37)	0.127	3.09 (0.72–13.25)	0.130	1.59 (0.24–10.74)	0.63	1.63 (0.24–11.08)	0.61
3 vs 1	8.47 (2.00–35.77)	**0.004**	8.55 (2.02–36.09)	**0.004**	0.17 (0.01–2.87)	0.22	0.21 (0.01–3.47)	0.28
ER (Negative vs Positive)	2.85 (1.42–5.75)	**0.003**	2.92 (0.15–5.89)	**0.003**	0.05 (0.00–1.50)	0.09	0.05 (0.00–1.47)	0.08
PR (Negative vs Positive)	2.55 (1.26–5.14)	**0.009**	2.60 (1.29–5.24)	**0.008**	7.94 (0.28–219.86)	0.22	7.81 (0.29–212.40)	0.22
HER2 (Negative vs Positive)	0.81 (0.44–1.47)	0.805	0.77 (0.42–1.39)	0.381	1.24 (0.66–2.35)	0.50	1.32 (0.70–2.51)	1.32
Ki67 (≤14% vs >14%)	0.46 (0.24–0.88)	**0.02**	0.41 (0.22–0.79)	**0.007**	0.65 (0.33–1.29)	0.22	0.57 (0.29–1.13)	0.57

T1: ≤2 cm; T2: >2 cm, ≤5 cm; T3: >5 cm. OS, overall survival time. DFS, disease-free survival time.

## DISCUSSION

A linear multi-step process of breast cancer development has been proposed in which ADH progresses to primary lobular and ductal carcinomas and ultimately results in invasive and metastatic tumors [18]. Exploring the biomarkers involved in these progresses, which has not been well reported thus far, will be beneficial for better diagnosis and prognosis of breast cancer. The phenomenon of NOP14 overexpression detected in the ADH, particularly the precancerous lesions, such as breast fibrocystic disease with atypical ductal hyperplasia, as well as lobular and ductal carcinomas *in situ*, indicated that NOP14 overexpression may be an applicable early warning biomarker for breast cancer.

Our results revealed the inhibitory effect of NOP14 in breast cancer development both *in vitro* and *in vivo* (Figure 2 and 3). Of note, NOP14 could suppress tumorigenesis *in vivo*, but has not significant effect on the proliferation ability *in vitro*. The seeming discrepancy between *in vivo* and *in vitro* results might be due to the regulation of tumor progression by tumor microenvironment *in vivo*, which is hardly recapitulated exactly *in vitro* [19]. Another possibility is that the inhibitory effect of NOP14 on the tumorigenesis might be a result from the restrain of breast cancer stem cells (CSC) (Supplementary Figure 3), which are the main source of tumor relapse and metastasis; however, the contribution of CSC to the proliferation of cultured cells may be negligible since CSCs occupy a pretty small portion in tumor cells. Moreover, it has been reported that CSCs grow much slower than non-stem cancer cells in xenograft mouse, indicating that high proliferation rate is not essential for tumorigenesis [20, 21]. This might be explained by the stem cell quiescence, a form of tumor suppression, which has been demonstrated previously [22].

Further investigations showed that *NRIP1*, which interacts with ERα [23], is the primary downstream gene of *NOP14*. The inhibitory effect of NRIP1 on carcinogenesis of colon cancer [17] and hepatocellular carcinoma [24] by up-regulating APC transcription and thus inhibiting the Wnt/β-catenin pathway, have been reported previously [17]. Most recently, low level of NRIP1 has been demonstrated as a marker of poor prognosis in chronic lymphocytic leukemia patients [25]. Interestingly, the inhibitions of cell migration and tumor metastasis by NOP14 were shown to be NRIP1-dependent, indicating that NRIP1 might be a major effector transducing NOP14 signaling (Figure 4). Consistent with these notions, NOP14 increased APC and β-catenin levels, as well as GSK-3β phosphorylation level in breast cancer cells; furthermore, NOP14 inhibited the entry of β-catenin into the nucleus of breast cancer cells (Figure 5). These results suggest that NOP14 exerts its functions on breast cancer cells through the Wnt/APC/β-catenin signaling pathway. In addition, *NRIP1* has been reported negatively correlated with breast cancer through a genome-wide association study (rs2823093; *P* = 1.1 × 10^−12^) [26], supporting the possible roles of *NOP14* and *NRIP1* in breast cancer development. Further investigations are pending to demonstrate whether the involvement of NOP14 in Wnt/APC/β-catenin signaling depends solely on NRIP1. Additionally, in ER-positive breast cancers, NOP14 increases the level of ERα via NRIP1, suggesting an interesting insight for future studies to improve the endocrinotherapy effects on breast cancer patients and lead to better prognosis.

Consistent with the tumor suppressor role of NOP14 in breast cancer, survival analysis suggested that low NOP14 expression is associated with poor patient outcome (Figure 6). Another analogous finding was recently made in ovarian cancer, where NOP14 overexpression in blood was associated with better survivals [27]. These suggest that NOP14 expression may be an applicable prognostic biomarker for breast cancer. In addition, an opposite effect of NOP14 has been reported that NOP14 can promote tumorigenesis and metastasis of pancreatic cancer cells [13], suggesting that the responsive genes downstream of NOP14 may differ among different types of cancer. Indeed, NRIP1 has been shown highly expressed in breast but rarely in pancreas according to RNAseq and SAGE database (http://www.genecards.org). Moreover, NOP14 interacts with PAXIP1 in yeast [10], but PAXIP1 was not found to be regulated by NOP14 at mRNA level in breast cancer (Supplementary Table S5).

In summary, we have showed that NOP14 might be a potential early warning biomarker for breast cancer; moreover, low NOP14 level is intimately associated with more invasive and metastatic breast cancers, and NOP14 suppresses breast cancer by inhibiting the Wnt/β-catenin pathways, possibly by up-regulating NRIP1; and furthermore, survival analysis has suggested that NOP14 level is a potential prognostic marker for breast cancer patients. These findings provide new insights into the development of targeted therapies against NOP14 and NRIP1 for breast cancer.

## MATERIALS AND METHODS

### Study approval

The institutional review boards at the Sun Yat-sen University Cancer Center (SYSUCC; Guangzhou, China) approved the study. All participants provided written informed consent. All animal experiments were conducted according to the institutional standard guidelines of Sun Yat-sen University. The animal approval number of the Guangdong Laboratory Animal Center is No.44007200009231.

### Samples and cell lines

Tumor and adjacent non-tumor specimens were collected from breast cancer patients diagnosed at the SYSUCC. The cell lines (MCF10A, MCF-7, BT474, MDA-MB-231, and MDA-MB-453) were purchased from American Type Culture Collection (ATCC, USA) and cultured in different media according to the instructions from ATCC.

### RNA extraction and qPCR

Total RNA was extracted using a Qiagen RNeasy Mini Kit (Qiagen, USA). In total, 2 μg RNA was reverse-transcribed into cDNA using Superscript III Reverse Transcriptase (Invitrogen, USA) according to the manufacturer's instructions. qPCR was performed on a CFX96™ Real-Time System (Bio-Rad, USA) using a Platinum^®^ SYBR^®^ Green qPCR Super Mix-UDG Kit (Invitrogen, USA). β-Actin was used as an internal control. The PCR conditions followed the instructions of the Platinum^®^ SYBR^®^ Green qPCR Super Mix-UDG Kit. Primer information is listed in Supplementary Table S3.

### Antibodies and WB analyses

Protein extraction and WB analyses were performed according to the standard protocol described previously [28]. Detailed information regarding the antibodies is listed in Supplementary Table S2

### Establishment of stable cell lines overexpressing NOP14

Full-length human *NOP14*cDNA was amplified by PCR, cloned into the pBABE-puro vector and then transfected into 293T packaging cells using Lipofectamine2000 (Invitrogen, USA) according to the manufacturer's instructions. The forward and reverse primer sequences were 5′-TTTGATATCGCCACCATGGCGAAGGCGAAGA AGGTCGGGG-3′, and5′-CCTTAATTAACTTGTCATCGTCGTCCTTGTAGTCTTATTTTTTGAACTTTTTCCTCTT-3′, respectively. Production of retroviral stocks and viral infections were performed according to the standard protocols from Addgene (http://www.addgene.org/). Cells transfected with empty vector were used as controls. Stable MDA-MB-231 and MCF-7 cells were selected in the presence of puromycin(0.5 μg/ml) for 1 week, and the surviving clones were pooled.

### Establishment of NOP14knockdown cell lines

shRNA sequences targeting NOP14 were obtained from Sigma-Aldrich. The oligo nucleotides were synthesized (Invitrogen, China) and then cloned into pLKO.1-puro. The MDA-MB-231 and MCF-7 cells stably transfected with shRNA were constructed according to the instructions provided by Addgene (http://www.addgene org/). The stable cells were selected against puromycin (0.5 μg/ml) for approximately one week, and the surviving clones were pooled. Cells transfected with a scrambled shRNA were used as controls.

### Tumorigenesis assays

For cell growth assays, the cells were seeded in 96-well plates at a density of 2 × 10^3^ cells per well, and cell growth rates were assessed using CCK-8 (Dojindo, USA). For the foci formation assay, 1 × 10^3^ cells were seeded in 6-well plates, and, after the cells were cultured for 3 weeks, the colonies were stained with crystal violet and counted. At least three independent experiments were performed for each assay; the results are shown as the mean ± standard deviation (SD). The xenograft model was established by the subcutaneous injection of 5 × 10^6^ MDA-MB-231 cells with *NOP14* overexpression, sh*NOP14*-4# targeting NOP14, scrambled shRNA or empty vector into the left dorsal flank of 5-week-old female nude mice. Tumor formation in nude mice was examined after 6 weeks. Detailed information is listed in Supplementary Table S5.

### Sphere formation assays

Sphere formation assays were performed according to the above-described procedures following previously reported protocols [29]. Briefly, 500 cells were seeded in 6-well plates coated with Ultra-Low Attachment Surface (Corning, USA), followed by culturing for 3 weeks in DMEM/F12 medium (Gibco, USA) supplemented with B27 (1:50;Gibco, USA), 20 ng/ml EGF (Life Technologies, USA) and 20 ng/ml basic FGF (Life Technologies, USA). The spheres were observed, imaged and counted under a microscope.

### Migration and invasion assays

For the migration assays, 200 μl of cells (4 × 10^4^ for MDA-MB-231 cells and 8 × 10^4^ for MCF-7 cells) was resuspended in DMEM and added to the upper chamber of Transwell plates (8-μm; Becton Dickinson, USA), and the lower chamber was filled with 700 μl of medium containing 10% FBS. The cell invasion assays were similar to the migration assays, except that the inserts were coated with Matrigel and an additional 2 × 10^4^ cells. After maintenance for 24 h, the cells were washed twice with PBS, fixed with methanol for 15 min, and again washed twice with PBS. The cells on the upper side of the filters were removed with cotton-tipped swabs and then stained with 0.5% crystal violet for 30 min. The inserts were air dried after washing in water. The cells on the bottom of the filters were counted in five randomly chosen fields using a 20× objective lens. The results are presented as the mean ± 3SD of three independent experiments.

### Metastasis assays

Metastasis assays were performed as previously described [30]. Specifically, MDA-MB-231 stable cell suspensions (1 × 10^6^ cells/200 μl) were injected into the tail veins of 4–6-week-old female nude mice. The mice were sacrificed at eight to ten weeks after inoculation (10 weeks for the overexpression group, 8 weeks for the knockdown group). The lungs were collected, weighed, fixed in 4% formaldehyde, embedded in paraffin and sectioned (5 μm). Serial sections were stained with hematoxylin-eosin, observed and imaged using different objective lenses. Detailed information is listed in Supplementary Table S5.

### Gene expression array

Total RNA from the stable cell lines was extracted and then analyzed by using the Prime View™ Human Gene Expression Array with more than 28, 869 transcripts at whole genome coverage (Cat No.: AFF-901085; Affymetrix, USA). The hybridization was done in CapitalBio (Beijing, China) following the manufactory's instructions. Briefly, an aliquot of 0.1 μg of total RNA was used to synthesize double-stranded cDNA and then to produce biotin-tagged cRNA using a Message Amp^™^ Premier RNA Amplification Kit. The resulting bio-tagged cRNA was fragmented into strands of 35–200 bases in length. The hybridization data were analyzed using the GeneChip Operating software (GCOS 1.4). An invariant set normalization procedure was performed to normalize the different arrays using DNA-Chip Analyzer. Downstream candidate genes were screened based on their differential expression compared with corresponding controls and based on their reported functions.

### IF staining

Cells were seeded onto coverslips in a 24-well plate. Forty-eight hours later, the cells were rinsed three times with pre-cooled PBS, fixed in 4% formaldehyde for 30 min, and washed with PBS three times for 5 min each. The cells were permeabilized with 0.2% Triton X-100 for 15 min, washed three times with PBST, blocked with 5% BSA for 1 h, and then incubated with primary antibody solution directed against β-catenin (1:100) at 4°C overnight. The next day, after the primary antibody solution was removed, the cells were washed three times with PBS, and then secondary antibody solution was added, followed by 1 h incubation at room temperature. After the cells were washed three times with PBS, they were stained with DAPI to label the nuclei. Finally, the cells were washed three times, and Anti-fade Mounting Medium was added. The images were acquired using a fluorescence microscope.

### Transient transfection

Full-length human *NOP14* or *NRIP1* cDNAs was, respectively, amplified by PCR, cloned into the pcDNA3.1 (+) expression vector (Invitrogen, USA), and then transfected into MDA-MB-231-shRNA-Ctl and MDA-MB-231-shNOP14 cells using Lipofectamine2000 (Invitrogen, USA) according to the manufacturer's instructions. As a control, pcDNA3.1 (+) empty vector was transfected into the corresponding cells. siRNA-NC and si*NRIP1* (RiboBio, China) was transfected into the MDA-MB-231-Vector (pBABE) and MDA-MB-231-NOP14 cells, respectively. The primer sequences were synthesized as previously described [17].

### TMA and IHC

Paraffin-embedded breast cancer slides from 363 cases, including 213 triple-negative and 150 luminal breast cancer cases, were obtained from SYSUCC. The patients in this retrospective study were diagnosed at SYSUCC between 1995 and 2008 and had no evidence of distant metastasis at the time of surgery. Tissue samples were obtained from the patients through curative surgical resection. All specimens were formalin-fixed, paraffin-embedded and constructed to the TMA [31, 32]. Clinical data including patient age at diagnosis, menstrual status, tumor size, lymph node status, pathological stage and follow-up status were retrospectively obtained from hospital medical records. IHC staining was performed as described previously [33]. High-pressure antigen retrieval was performed for 3 min, and the primary antibody concentration was 1:100. Ki67 staining results were reviewed by two independent pathologists.

### Software and statistical analysis

SPSS 17.0 was used for all data analyses (SPSS, USA). The relative expression levels of the targeted genes were quantified and analyzed using GraphPad Prism 5.0 software (GraphPad Software Inc., USA). Student's *t*-test was used for comparative analyses. Correlations were assessed by Pearson's chi-square test. Survival analyses were performed using Kaplan-Meier plots and log-rank tests to evaluate the role of NOP14 in breast cancer prognosis. Univariate and multivariate survival analyses were performed using the Cox proportional hazards regression model to test whether NOP14 is an independent prognostic factor. All statistical tests were two-tailed, and *P* < 0.05 was considered significant. DFS was defined as the interval from the first treatment for breast cancer to the first recurrence (locoregional relapse, distant metastasis, or contralateral breast). OS was calculated as the period from the date of diagnosis to the date of death from any cause or last follow-up.

## SUPPLEMENTARY MATERIALS, FIGURES AND TABLES


