# Supplementary material for: NOP14 suppresses breast cancer progression by inhibiting NRIP1/Wnt/β-catenin pathway
Source: Oncotarget. 2015 Jul 13;6(28):25701–14. doi: 10.18632/oncotarget.4573 (PMC4694860; doi:10.18632/oncotarget.4573)
Supplement: Supplementary file 1 [file oncotarget-06-25701-s001.pdf]

SUPPLEMENTARY MATERIALS

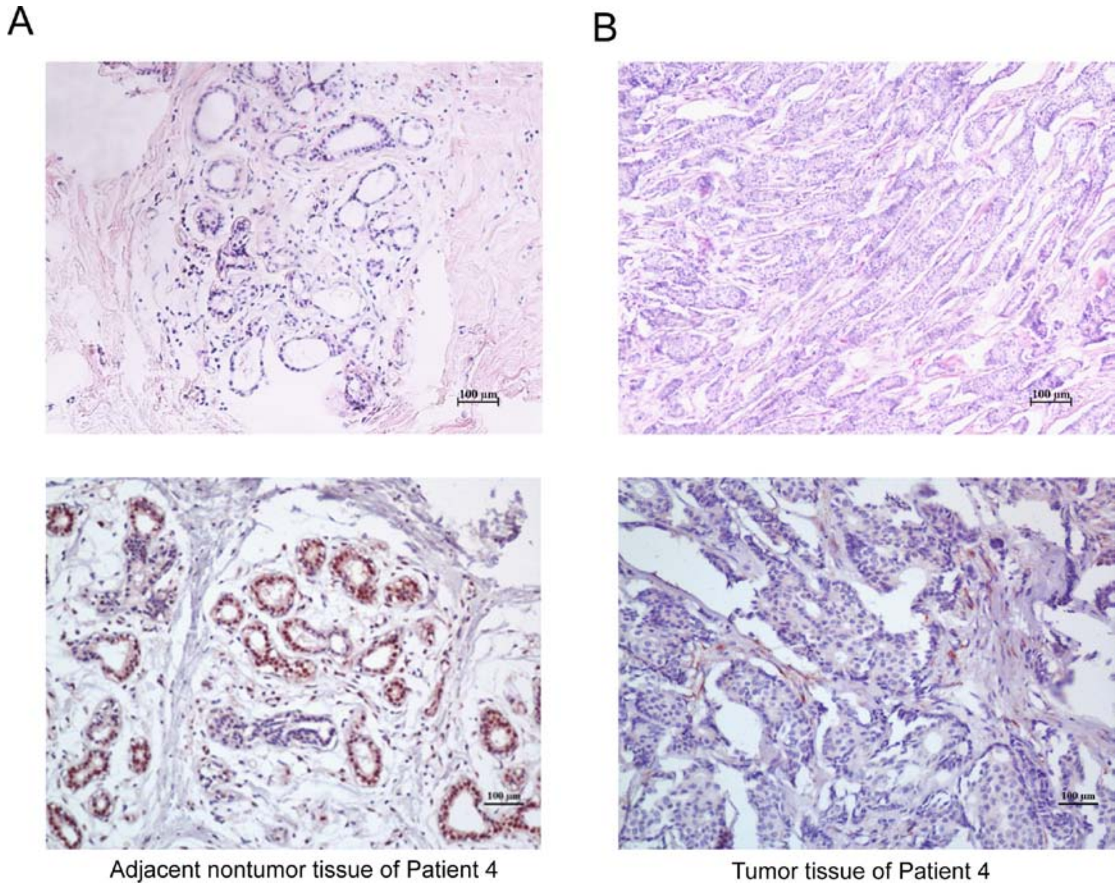

Supplementary Figure S1: Expression of NOP14 in a patient. A. Expression of NOP14 in adjacent nontumor tissue, which presents fibrocystic mammary gland disease. **B.** Expression of NOP14 in tumor (invasive ductal carcinoma) tissue. The two different histopathological tissues are on the same slide and NOP14 expression is tested by IHC. HE staining was showed in the upper panel.

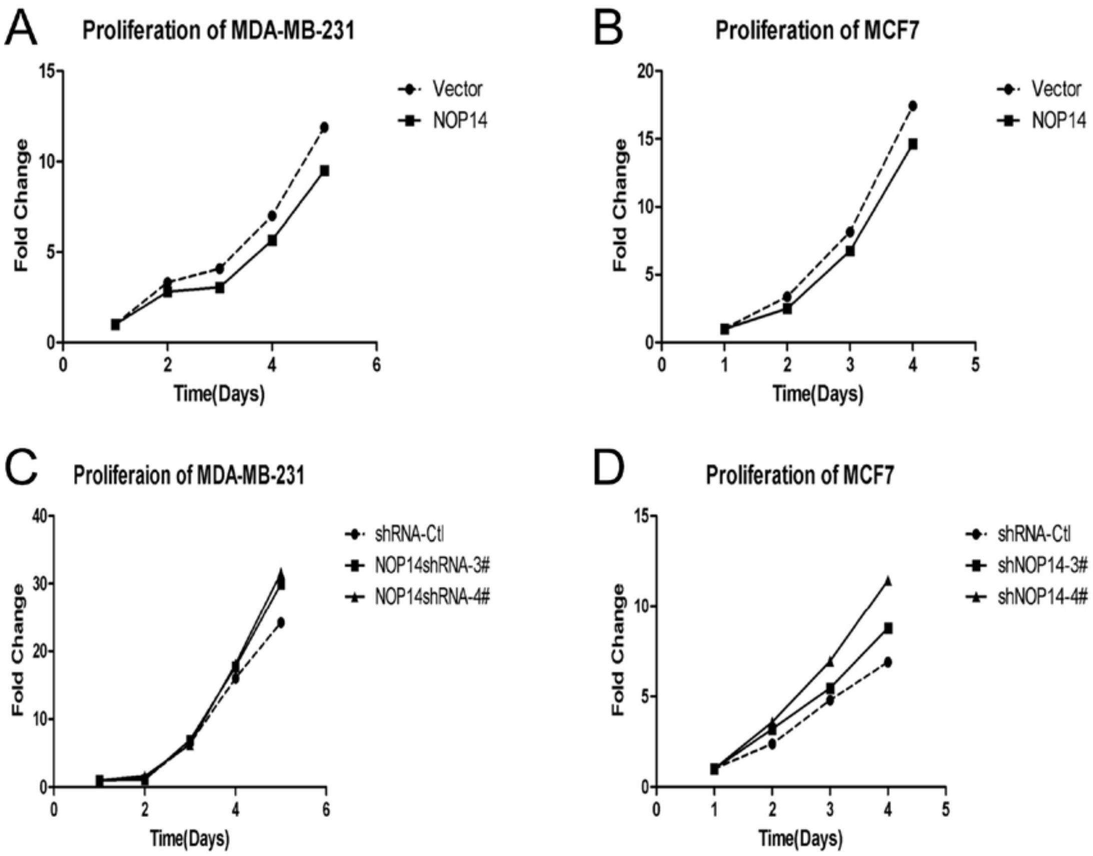

Supplementary Figure S2: Proliferation assays of NOP14 in breast cancer cell. A, B. Cell proliferation rates of NOP14-expressed and empty vector stable cells were assayed by CCK-8. The results are expressed as the mean ± 6SD of three independent experiments. **C, D.** Cell proliferation rate of NOP14 silencing and scrambled cells was assayed by CCK-8. The results are expressed as the mean ± 6SD of three independent experiments.

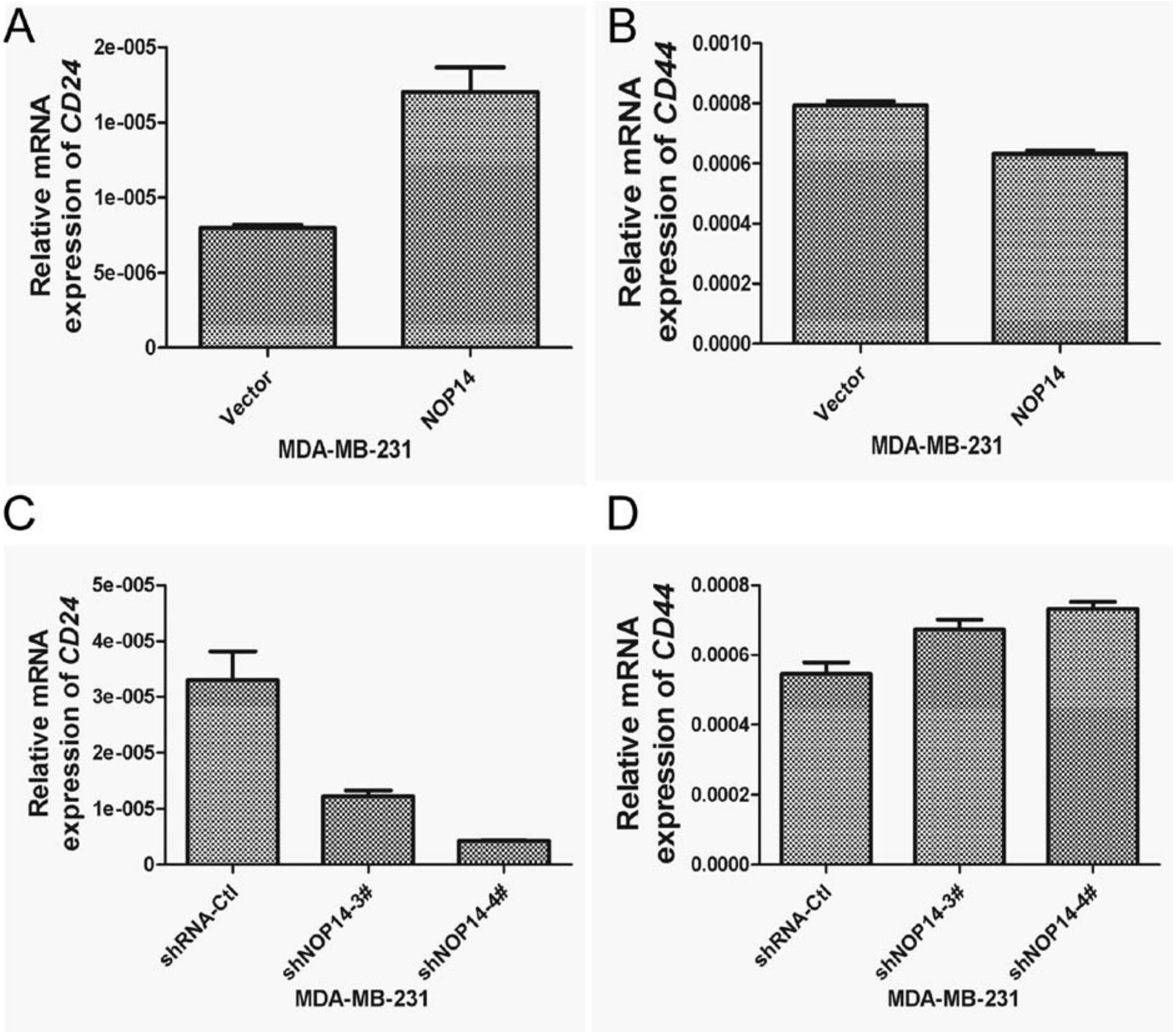

Supplementary Figure S3: qPCR assays showing relative expression levels of CD24 and CD44 in NOP14-expressed (A and B) and NOP14-silenced (C and D) and the corresponding controls. β-Actin was used as reference.

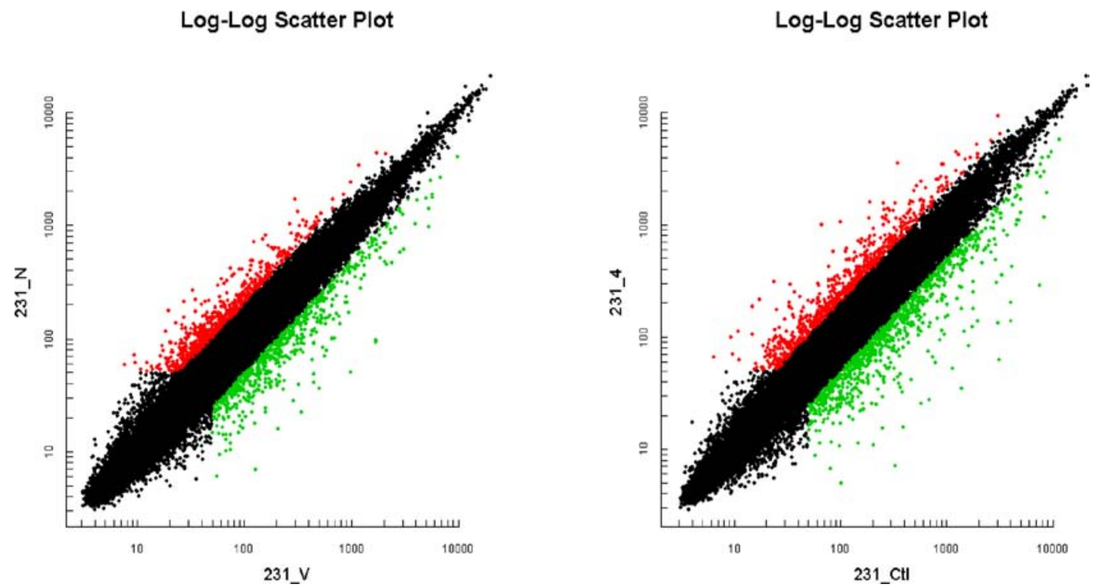

Supplementary Figure S4: Representative scatter plot of Gene Expression Array with NOP14 -expressed and NOP14-silenced compared with corresponding control cells. 231 is short for MDA-MB-231, N, V is short for NOP14 and Vector respectively; Ctl and 4 means scrambled shRNA Control and shNOP14–4# separately. Red dots stand for upregulated genes while green dots represents downregulated genes**.**

Supplementary Table S1: The expression of NOP14 in different breast tissues

Kinds of breast tissues	No. of cases	Condition of NOP14 expression
Normal breast tissue	7	(+)
Fibrocystic mammary gland disease	95	(+++)
Breast carcinoma in situ	42	(+++)
Invasive breast carcinoma	254	(-)
Metastatic breast cancer	63	(-)

Supplementary Table S2: Relative gene expression in Gene Expression Array

Gene Name	NOP14/Vector		shNOP14–4#/shRNA-Ctl	
	MCF7	MDA-MB-231	MCF7	MDA-MB-231
NOP14	2.1	5.6262	0.3565	0.6517
NRIP1	1.4561	4.9566	0.4208	0.2705
LSR	1.1685	1.5012	0.9478	0.5396
ZNF354A	1.0615	1.5303	0.9440	0.4896
ZNF555	1.0645	1.5486	0.6276	0.5172
DAPK2	1.4636	1. .2276	0.7619*	0.9246
CRNDE	1.0281	1.1043	0.9750	0.7131
SCNN1B	1.3595	0.7999*	0.9165	0.6971*
AMELX	1.5411	1.1115	0.8742	0.7325
C6orf145	1.6314	1.0653	0.6161*	0.5707
SEPP1	1.1301*	1.4093	0.6166*	0.9952
CSF2	1.1579	1.6255	0.8363	0.1399
NCEH1	0.9602*	0.3362	1.547	2.2349
CALU	0.9254	0.476	1.4766	2.3579
HSP90AA4P	0.9663	0.6335	1.0143	2.6998
GALNT10	0.5176	0.7483*	1.0491	2.0158*
RRM2	0.8959	0.6889	1.4890	3.4623
CCND1	0.8871	0.6544	1.5253	1.2067
PCYOX1L	0.8278	0.8172	1.2396	1.2159
POLD2	0.9162	0.9732	1.1304	1.1132
SRA1	0.6727	0.9784	1.0628	1.2754
ODC1	0.7703	0.9516	1.4138	1.2487

*the level of gene expression is very low, the ratio might be unreliable.

Supplementary Table S3: Tumor weight and wet weight of lungs of nude mice model

Tumor weight (mg)				Wet weight of lungs (mg)			
Vector	NOP14	shRNA-Ctl	ShNOP14–4#	Vector	NOP14	shRNA-Ctl	ShNOP14–4#
477	/	60	400	603	395	253	393
369	/	54	392	502	373	247	319
271	/	25	172	433	323	192	298
71	/	11	85	411	323	190	281
70	/	9	75	403	315		273
64	/	8	73	*	277		271
57	/	/	72	*	271		262
39	/			*	256		
34	/			*	229		
20	/						
10							

/, no tumor formed; *mouse died during the observation.

Supplementary Table S4: Primer sequences of qPCR

Gene Symbol	Forward Sequence	Reverse Sequence
NOP14	GAAGTCCCGGAAAGAGCTGA	TGCATCGGGCTTGGGTTTTT
NRIP1	AATGTGCACTTGAGCCATGATG	TCGGACACTGGTAAGGCAGG
APC	TAGGGCTAGGCAGGCTGTG	CTGCACCAATACAGCCACAT
β-Actin	AGATGTGGATCAGCAAGCAGGAG	CGCAAGTTAGGTTTTGTCAAGAAAGG
CD24	GCGCATTTTGCAGTCTGAGT	CACTGGAATAAATCTGCGTGGG
CD44	TCCCTCCGTCTTAGGTCACT	TATTCAAATCGATCTGCGCCA

Supplementary Table S5: Antibodies presentations

Short name	Full name	Company	Cat. NO.
NOP14	Anti-Nucleolar protein 14 antibody	abcam	ab72741
NRIP1	RIP140 Antibody	Santa Cruz Biotechnology	sc-8997
GSK-3β	GSK-3β (27C10) Rabbit mAb	Cell Signaling Technology	#9315
pGSK-3β	Phospho-GSK-3β(Ser9) (5B3) Rabbit mAb	Cell Signaling Technology	#9323
β-Catenin	β-Catenin (D10A8) XP® Rabbit mAb	Cell Signaling Technology	#8480
ERα	Estrogen Receptor α (D8H8) Rabbit mAb	Cell Signaling Technology	#8644
β-Actin	β-Actin (13E5) Rabbit mAb	Cell Signaling Technology	#4970
GAPDH	Anti-GAPDH antibody	abcam	ab75479
Flag	Anti-FLAG M1 monoclonal antibody	Sigma-Aldrich	F3040
